# Barriers and enablers to exercise prehabilitation before breast cancer surgery in an Australian regional health service: patient and clinician perspective

**DOI:** 10.1007/s00520-025-09261-8

**Published:** 2025-02-21

**Authors:** April Chiu, Sarah Huntly, Breanna McPhee, Molly Branson, Matthew Wallen, Declan Hennessy

**Affiliations:** 1 Barwon Health, Geelong, Victoria Australia; 2 Institute of Health and Wellbeing, Federation University Australia, Ballarat, Victoria Australia; 3 Caring Futures Institute, College of Nursing and Health Sciences, Flinders University, Adelaide, South Australia Australia; 4 School of Exercise and Nutrition Sciences, Faculty of Health, Deakin University, Burwood, VIC Australia

**Keywords:** Breast cancer, Exercise, Prehabilitation, Preoperative, Supportive care

## Abstract

**Purpose:**

To identify barriers and enablers of an exercise-based prehabilitation service for surgical patients with breast cancer and medical professionals in a regional healthcare setting.

**Methods:**

A cross-sectional survey was distributed to patients with breast cancer and medical professionals from regional populations. Surveys included closed and open-ended responses. A chi-square goodness of fit test with a Fisher’s exact correction was used for quantitative analysis of the frequencies of barriers and enablers within groups. Content analysis was used for open-ended responses.

**Results:**

Twenty-five patients and 14 clinicians participated. Patients identified psychological barriers as the lowest concern to exercise prehabilitation (*χ*^2^(9, *n* = 23) = 21.78, *p* = .011). No other patient barriers were statistically significant. Fifty-nine percent of patients expressed interest in participation in exercise prehabilitation, with 40% citing personal fitness benefits as the leading enabler. Clinicians identified time constraints as a barrier (*χ*^2^(2, *n* = 13) = 8.00, *p* < .05), with challenges integrating prehabilitation into pre-surgery timelines. Clinicians indicated electronic referral methods and information would be enablers for prehabilitation.

**Conclusion:**

The study underpins the need for integrating exercise professionals into preoperative teams to address exercise prehabilitation within limited time frames. Patients do not describe psychological barriers and instead report motivation to improve fitness as an enabler of prehabilitation. Clinicians report time constraints in pre-operative appointments as a barrier to prehabilitation. Implementing electronic referral methods alongside traditional approaches may enhance prehabilitation delivery for patients receiving breast cancer surgery. Future research should leverage these findings for prehabilitation referral and program design.

**Supplementary Information:**

The online version contains supplementary material available at 10.1007/s00520-025-09261-8.

## Introduction

### Background

Breast cancer is the most frequently diagnosed cancer among Australians [1] and predominantly involves surgical intervention for its treatment [1]. With advancements in detection and treatment, a large number of Australians continue to live with breast cancer, with 5-year survival rates at 92% between 2013 and 2017 [1].

Post-surgical complications remain problematic in this cohort. Axillary web syndrome and brachial plexopathy are most common and contribute to increased pain levels, reduced shoulder and neck range of motion, lymphoedema, and fatigue [2, 3]. These complications significantly impact activities of daily living and emotional, social, physical, and economic well-being [2]. Moreover, health-related quality of life (HRQoL) among patients with breast cancer can suffer for up to a decade post-treatment due to the enduring effects of surgery and adjuvant treatments [3, 4].

Exercise delivered across the breast cancer treatment continuum reduces treatment-related side effects and improves overall well-being [5]. Efforts aimed at enhancing post-operative musculoskeletal function and HRQoL have focused primarily on post-operative rehabilitation when many impairments have already become chronic [10]. Recently, there has been a shift towards pre-operative intervention—termed ‘prehabilitation’ which involves comprehensive assessment and targeted interventions before major oncological treatments, like surgery [6]. Prehabilitation interventions can include individualised exercise programs, respiratory education, nutritional optimisation, psychological support, behaviour change, and medical optimisation [7]. Currently, pre-operative exercise is the predominant prehabilitation modality researched in supportive cancer care [8–10]. Specific to breast cancer, exercise prehabilitation improves pre-operative cardiorespiratory fitness, decreases post-operative pain, improves shoulder range of motion and function, and is associated with an improved sense of post-operative well-being compared to no prehabilitation [5].

Despite evidence supporting exercise prehabilitation before cancer surgery, its adoption as part of standard care is limited [11]. The reasons for underutilisation of prehabilitation include lack of interest, time constraints, and limited availability before imminent surgery [12]. Understanding why these barriers exist is crucial, especially in regional settings, to enhance service accessibility and align with evidence-based optimal care pathways [13].

At Barwon Health, a regional health service in Victoria, Australia, there is an established care pathway for patients with breast cancer to access exercise prehabilitation services. Despite this established care pathway, the uptake of exercise prehabilitation remains low. In 2020, an internal audit showed only two out of 198 breast cancer surgery patients at the regional health service were referred for prehabilitation before surgery, with referrals typically occurring post-surgery. By soliciting input from key stakeholders, including healthcare professionals and potential service users, we seek to explore barriers and enablers for exercise prehabilitation in an attempt to improve care pathways, align with evidence-based care, and enhance long-term patient outcomes [13].

### Objectives

The primary aim of this study was to explore the barriers and enablers for patients and clinicians of an established prehabilitation pathway before breast cancer surgery.

## Methods

### Study design

A STROBE-compliant prospective, cross-sectional survey was administered to patients and clinicians to identify barriers and enablers for prehabilitation before breast cancer surgery [14]. Ethical approval was granted through the Barwon Health Human Research Ethics Committee (22/38). All participants gave written and informed consent prior to participating in the study.

### Setting

This study was conducted at the Barwon Health’s University Hospital Geelong and Andrew Love Cancer Centre (ALCC). Barwon Health and the ALCC provide public health care, including radiation, medical oncology, and surgical services to rural and regional populations of approximately 400,000 people [15]. The closest metropolitan centre to ALCC is Melbourne (74 km). ALCC has access to Barwon Health allied health services, including a specialist cancer exercise service and associated rehabilitation facility approximately 2.3 km from ALCC. A convenience sampling method was utilised to recruit patients with breast cancer [16]. All patients were recruited from these sites through outpatient clinics, and all staff involved in data collection were Barwon Health employees.

### Participants

#### Patients with breast cancer

Adult patients (> 18 years) proficient in English were eligible to participate in the study if they (a) received surgical intervention for the treatment of histologically confirmed breast cancer, (b) were over the age of 18, (c) were proficient with the English language, and (d) were provided with written information on the study and a link to the online survey by the ward Physiotherapist within 24 h following their breast cancer surgery, during their inpatient stay. All patients receiving surgery for breast cancer had an initial appointment at the ALCC prior to surgery, at which the prehabilitation service could have been offered by any ALCC clinical staff to the patients. Patients with a concurrent diagnosis of another cancer type were excluded from participation.

#### Clinicians

Medical professionals, including medical oncologists, radiation oncologists, anaesthetists, breast surgeons, breast care nurses, tumour stream coordinators, and registered nurses working in breast cancer services at ALCC were invited to participate. Allied Health professionals are not routinely involved in pre-operative care to patients with breast cancer and thus were not included in the survey population. Clinicians were contacted via email with a link for the online Participant Information and Consent form and survey. There were no exclusion criteria for clinicians to participate.

### Data sources and measurement

Recruitment occurred from February 2023 until September 2023. Surveys were administered via secure online data capture software (REDCap), and where unable to do this, hard copies of surveys were provided to participants and manually entered into the database as per best practice recommendations [17, 18].

The research team constructed surveys based on Nadler et al. and adapted them to the cancer context based on Elbourne et al. [19, 20].

### Quantitative variables

The patient participant survey included closed questions regarding participant demographic and exercise engagement (10 items) and perceptions of exercise-based appointments (7 items), as well as barriers (34 items) and enablers for participation in exercise-based prehabilitation prior to breast cancer surgery (6 items). All items were rated on a 5-point Likert scale ranging from ‘strongly disagree = 1’ (1) to ‘strongly agree = 5’. Free-text options were also provided to gather any additional information. Patient participants were sent an electronic reminder (SMS or email) 2 weeks after initial contact with the ward Physiotherapist to complete the survey.

The clinician survey captured information on demographics (2 items), barriers (12 items), and enablers (11 items) to exercise-based prehabilitation prior to breast cancer surgery. All items were also rated on the same 5-point Likert scale from ‘strongly disagree = 1’ to ‘strongly agree = 5’ and free-text options were also provided to gather any additional information. See ‘Supplementary Material’ for the Patient Participant Survey and Clinician Survey.

### Statistical methods

#### Quantitative data

Clinician and patient data were analysed using SPSS statistical software (Version 20.1) by an independent investigator who had no involvement in study recruitment or data collection. Patient questionnaire data were segmented into six analytical categories—Practical, Social, Physical, Psychological, Knowledge-based, and Motivational barriers. These categories were based on current literature findings to further aid in the interpretation of results and provide a comprehensive understanding of the barriers encountered [21–25]. Clinician questionnaire data was analysed at an item-by-item level. Descriptive statistics were explored, where continuous data was presented as mean ± standard deviation or median (interquartile range) where appropriate and categorical data was presented as a count (percentage of the cohort). A chi-square goodness-of-fit test with a Fisher’s exact correction was used to examine the deviation of observed frequencies in the data from an expected equal distribution within small samples with low cell counts [26, 27]. All available data was included in the analysis. A *P*-value < 0.05 denoted statistical significance.

#### Qualitative data

Where possible, to elucidate patient and clinician perspectives on barriers and enablers of prehabilitation in breast cancer, written responses were examined using content analysis [28]. Due to limited qualitative data volume, qualitative results should be interpreted with caution.

## Results

### Participants

A total of 25 female patients with breast cancer and 14 clinicians participated in the study. Complete survey responses were provided by 92% and 92.8% of patients and clinicians, respectively.

### Descriptive data

Demographic information is presented in Tables 1 and 2.

**Table 1:** Demographics of patients

Outcome	*N* (%)
Age (years)	
18–24	0 (0%)
25–34	2 (8.3%)
35–44	2 (8.3%)
45–54	5 (20.8%)
55–64	6 (25%)
65 +	9 (37.5%)
Identifies as Aboriginal or Torres Strait Islander	
No	24 (95.8%)
Yes	1 (4.2%)
Distance from treating centre (km)	
0–5	4 (16.7%)
5–10	7 (29.2%)
20–30	11 (45.8%)
30–40	1 (4.2%)
40–50	0 (0%)
50 +	1 (4.2%)
Self-report regular exercise prior to surgery	
No	14 (58.3%)
Yes	10 (41.7%)

**Table 2:** Demographics of clinicians

Outcome	*N* (%)
Medical occupation	
Anaesthetist	6 (42%)
Surgeon	4 (29%)
Breast care nurse	3 (21%)
Medical oncologist	1 (7%)
Clinical experience in cancer (years)	
0–2	2 (14%)
2–5	1 (7%)
5–10	5 (36%)
10–15	3 (21%)
15–20	1 (7%)
20 +	2 (14%)
Clinical focus	
Breast	12 (86%)
Prostate	5 (36%)
Gastrointestinal	5 (36%)
Genitourinary	3 (21%)
Gynaecological	2 (14%)
Head and neck	4 (29%)
Lung	4 (29%)
Skin	5 (36%)
Other	2 (14%)

### Outcome data

#### Patients

Practical, social, and motivation barriers to exercise prehabilitation were rated between “disagree” and “neither agree nor disagree”, whereas diagnosis/physical was rated as “disagree”. Only knowledge barriers were identified by patients as “neither agree nor disagree”. A statistically significant finding indicated that only a small percentage of patients identified psychological factors as barriers to prehabilitation (*χ*^2^, (9, *n* = 23) = 21.78, *P (exact)* = 0.011). Patients rated psychological factors as between “strongly disagree” and “disagree” (M = 1.96, SD = 0.19) as a barrier to exercise prehabilitation prior to surgery (See Table 3 for further details).

**Table 3:** Patient barriers to prehabilitation

	Median (SE)	Mean (SD)	Min–max	*χ*^2^
Practical	2.25 (.16)	2.19 (0.78)	1–3.4	6.96
Social	2.33 (.20)	2.31 (0.94)	1–4	8.74
Diagnosis and physical	2.00 (.20)	2.19 (0.94)	1–4	8.83
Psychological	1.96 (.19)	1.96 (0.92)	1–3.75	21.78*
Knowledge	3.00 (.25)	2.65 (1.21)	1–5	8.65
Motivation	2.5 (.21)	2.30 (1.01)	1–4.33	11.57

*p < .05; 1 = strongly disagree; 2 = disagree; 3 = neither agree nor disagree; 4 = agree; 5 = strongly agree

Free-text response qualitative content analysis revealed that 24% of patient participants reported engaging in exercise prior to diagnosis and before surgery. Additionally, 12% of patient participants reported that clinicians recommended exercise prior to surgery, and 67% of those adhered to these recommendations:

*“Breast care nurses were very proactive in advocating exercise prior and after surgery”*

*“Received info at time of diagnosis which reinforced decision to continue exercise.”*

Furthermore, quantitative participant survey responses demonstrated over half (59%) of patients indicated that they would be interested in participating in a tailored exercise and education program in the lead-up to their surgery. The most common interest in prehabilitation was *personal fitness benefits* (40%), followed by *to learn more about how to prepare for surgery* (36%).

Patients’ quantitative survey responses also indicated that the most useful resources that could be made available to them were written information about exercise and breast cancer (80%), followed by written information about pre-operative exercise and education (60%).

#### Clinicians

### Time to referral and surgery

Of the clinician sample (*n* = 13), results from Fisher’s exact goodness-of-fit test indicated that there was a statistically significant percentage of clinicians who rated limited time with patients as a barrier to prehabilitation, *χ*^2^, (2, *n* = 13) = 8.00, *p* < 0.05. Clinicians rated insufficient time during a pre-operative patient visit as the strongest barrier to referring patients for prehabilitation prior to surgery.

Qualitative clinician responses demonstrated 23% of clinicians referred to the amount of “time” they were allocated with the patient in clinic, as well as the shortened time frames from initial appointment to surgery were a barrier to exercise prehabilitation (See Tables 4 and 5 for further details).

**Table 4:** Clinician barriers to recommending exercise

Descriptive statistics and test statistics for Fisher’s exact test of clinician barriers to prehabilitation				
	Median (SE)	Mean (SD)	Min–max	*χ*^2^
Lack of training	2.0 (.36)	2.23 (1.30)	1–5	4.31
Pre-op exercise irrelevant	1.0 (.18)	1.46 (0.67)	1–3	5.69
Pre-op exercise unsafe for patient	2.0 (.28)	2.00 (1.00)	1–4	2.92
Insufficient time with patient	4.0 (.24)	3.46 (0.88)	2–4	8.00*
Lack of knowledge regarding referrals	3.0 (.35)	2.92 (1.26)	1–5	2.00
Exercise futile	2.0 (.24)	1.83 (.84)	1–4	4.50
Fear of inciting patient guilt	2.0 (.22)	1.85 (.80)	1–4	5.69
Influence from others to rest	3.0 (.27)	2.85 (.99)	1–4	2.08
Patient has previously refused support	3.0 (.20)	2.77 (.73)	2–4	2.00
Scientific support for prehabilitation unconvincing	2.0 (.14)	1.54 (.52)	1–2	.08
Insufficient time for patient to engage in prehabilitation	3.0 (.24)	3.08 (.86)	2–4	.15

*p = < .05; 1 = strongly disagree; 2 = disagree; 3 = neither agree nor disagree; 4 = agree; 5 = strongly agree

**Table 5:** Clinician enablers to recommending exercise

	Median (SE)	Mean (SD)	Min–max	*χ*^2^
Written information	4.0 (.14)	4.46 (0.52)	4–5	0.08
Clinician education	4.0 (.28)	4.0 (1.00)	2–5	2.92
Paper referral information	4.0 (.27)	3.85 (0.99)	2–5	6.39
Electronic referral information	4.0 (.23)	4.00 (0.82)	2–5	10.08*
Automatic referral process	4.0 (.28)	3.77 (1.01)	2–5	3.31
Posters	4.0 (.29)	3.92 (1.04)	2–5	4.54
Information provided outside of clinic time	4.0 (.30)	3.83 (1.03)	2–5	4.67
Patient handout information	4.0 (.13)	4.31 (0.48)	4–5	1.92
Physiotherapist/EP on clinical team	5.0 (.23)	4.50 (0.80)	3–5	6.00
Physiotherapist/EP in pre-op appointments	5.0 (.28)	4.23(1.01)	2–5	6.39

*p = < .05; 1 = strongly disagree; 2 = disagree; 3 = neither agree nor disagree; 4 = agree; 5 = strongly agree

### Additional enablers

Regarding additional enablers to exercise prehabilitation, 77% of clinicians reported they “agree” or “strongly agree” that the inclusion of a Physiotherapist or Exercise Physiologist in the preoperative appointments prior to surgery would be beneficial. Furthermore, 62% “strongly agree” that it would be beneficial to have those professions available in the team. Seventy-seven percent of clinicians rated “agree” or “strongly agree” that education sessions provided to clinicians about exercise would be helpful. Sixty-nine percent agreed that having exercise information provided outside of medical clinic time would be of benefit.

Results from the Fisher’s exact goodness-of-fit test indicated that there was a statistically significant percentage of clinicians who agreed electronic and web-based forms could be helpful with referral information, *χ*^2^, (2, *n* = 13) = 10.08, *p* = 0.02.

## Discussion

### Key results

This study explored the perceptions of both patients and healthcare providers on the barriers and enablers influencing the uptake of an exercise prehabilitation program for patients with breast cancer at a regional healthcare facility. Our findings identified that for patients, psychological barriers, including the fear of worsening their cancer or symptoms, did not serve as barriers to prehabilitation engagement. Limited time for clinicians during pre-operative consultations for discussions about exercise was a significant barrier to prehabilitation. However, written documentation was considered a potential significant enabler of prehabilitation.

### Barriers and enablers to prehabilitation—clinicians

Limited time with patients in appointments was reported by clinicians as a significant barrier to discuss exercise prehabilitation. This reflects current evidence showing time constraints as a major barrier to referral to general exercise in cancer patients by clinicians and for uptake of exercise prehabilitation by patients with breast cancer [7, 12, 29]. The current Optimal Care Pathway standards in Australia for managing breast cancer require patients to receive their surgical intervention within approximately 2 weeks from diagnosis [30]. While this limited time frame suggests that prehabilitation programs and referral pathways typically implemented in other cancer streams may not be feasible, it is worth noting that some studies in lung cancer have shown that a 2-week period may be sufficient to improve exercise capacity and reduce postoperative pulmonary complications (PPC). However, the applicability of these findings to breast cancer prehabilitation remains uncertain, given the differences in cancer types and treatment protocols [31, 32].

Written information was considered an enabler for referral to prehabilitation and electronic methods of referral and information distribution were rated highly. This finding partially conflicts with current evidence regarding non-cancer-specific health education, which shows that only providing printed educational materials has limited effects on general health outcomes [33]. However, the combination of in-person and written (hard-copy or digital) patient education has been found to be beneficial in cancer care [33]. The dynamic between clinician preference and patient enablers should be further investigated [34].

The inclusion of an exercise professional (e.g., Physiotherapist or Accredited Exercise Physiologist) within the clinical team and as part of routine pre-operative appointments was identified as an enabler of exercise prehabilitation, albeit not statistically significant. However, recent evidence from a systematic review and meta-analysis on exercise-based cancer rehabilitation via telehealth highlights the potential of integrating these professionals in a remote, supervised setting [35]. Additionally, adopting a systems-thinking approach suggests that there are new opportunities to optimise the model of care by embedding cancer patients into existing exercise programs, such as cardiac rehabilitation, particularly when service availability is limited [36]. This approach could help circumvent barriers related to time and resource constraints. Moreover, changes in referral systems, guided by a systems-thinking perspective, could streamline the process, ensuring that cancer patients are effectively integrated into these multidisciplinary exercise programs. Therefore, future research should explore the efficacy of such an altered model of care, which could enhance patient outcomes and improve the sustainability of prehabilitation practices.

### Barriers and enablers to prehabilitation—patients

Patients reported that psychological factors, including the fear of exacerbating cancer or other medical conditions, embarrassment, or fear of injury, were not barriers to participation in exercise prehabilitation prior to breast cancer surgery. This aligns with previous research reiterating that patients are not limited from engaging in rehabilitation by emotional or psychological concerns [19]. Patients reported no discouragement by clinicians from exercise and 12% of patient participants were instead encouraged to be physically active while awaiting surgery [29]. This aligns with the findings of recent literature, which emphasise the importance of early and integrated referrals to exercise professionals as part of a multidisciplinary approach to cancer care [37]. Qualitative survey responses further reinforced that medical staff supported exercise; patients noted being encouraged by clinicians to maintain their exercise routines following diagnosis. This support reflects broader consensus on the essential role of physical activity in cancer care, as outlined in expert recommendations for standardised referral practices to ensure all patients receive consistent and effective guidance on maintaining physical activity during treatment [37].

In this study, patient responses regarding practical factors did not demonstrate any significant findings. However, previous research finds practical factors such as transport, lack of time, and lack of convenient facilities were significant barriers to prehabilitation [7, 38]. Interestingly, no statistically significant barriers were found in other subcategories (social, physical, knowledge, motivation). This may be attributed to the preserved physical status and exercise habits of patients who have yet to receive surgery or any other cancer treatments [8].

### Strength and limitations

To our knowledge, there have been no studies investigating barriers and enablers to breast cancer prehabilitation exercise in any regional healthcare setting. The findings from this research not only bridge the gap between clinicians and patients regarding service engagement but also highlight the collaboration between medical, nursing, and allied health teams. By demonstrating that the lack of time for clinicians is a significant barrier to discussing prehabilitation, this study has identified that an alternative model for prehabilitation discussion and referral that does not require further clinician time may need to be explored. Furthermore, by revealing a lack of psychological barriers from patients to participating in prehabilitation, this research will help direct further investigation towards non-psychological barriers, which may be greater limiting factors. These findings inform the delivery of best practices with consumer consultation, which underpins any potential service delivery changes in the regional healthcare setting.

A significant limitation of this study is the limited sample size of participants for both clinicians and patients, which reduces the power and generalisation of these findings. Low enrolment numbers for this study may be due to rapid discharge time frames from the acute ward prior to the opportunity for recruitment and limited time for acute Physiotherapists to recruit patients due to clinical case load. Additionally, by introducing the research study to patients only 1 day after their surgery and during their inpatient stay, uptake for survey completion may have also reduced as patients may have been overwhelmed with their acute stage of recovery. As a result, this study is unable to draw significant conclusions as the overall data may be overly impacted by a small subset of findings. Furthermore, self-selection bias may be present as patient participants who were more likely to complete the survey may have had a more positive view of exercise or prehabilitation. The exclusion of allied health professionals from the clinician participant cohort is also a limitation as allied health professionals are essential in the delivery of prehabilitation and could provide valuable insights into barriers and enablers to this service. Although allied health professionals are not routinely involved in the prehabilitation service at this health service, their feedback would be valuable in future studies looking at a broader clinical context.

Future research should investigate the feasibility of alternative referral pathways to regional prehabilitation programs as informed by findings gathered from this study. Further research should also include a larger sample size to improve the strength of findings for generalisation to broader regional and rural settings. Additionally, a broader range of variables for patient participants such as economic status and caregiver responsibilities could be investigated as potential barriers and enablers.

### Interpretation and generalisability

The findings of this research will underpin the design of future referral pathways and supportive care programs involving prehabilitation for breast cancer surgery. The study findings indicate that including exercise professionals and Physiotherapists as part of the preoperative team may benefit addressing prehabilitation needs within the limited time frame from diagnosis to surgery. Furthermore, electronic information and referrals for clinicians and patients in conjunction with face-to-face input may be effective in delivering prehabilitation to patients receiving surgery for breast cancer.

## Supplementary Information

Below is the link to the electronic supplementary material.

Supplementary file1 (PDF 57 KB)

Supplementary file2 (PDF 52 KB)

## Data Availability

No datasets were generated or analysed during the current study.

## Code availability

N/A.
