# Supplementary material for: Barriers and enablers to exercise prehabilitation before breast cancer surgery in an Australian regional health service: patient and clinician perspective
Source: Support Care Cancer. 2025 Feb 21;33(3):211. doi: 10.1007/s00520-025-09261-8 (PMC11845435; doi:10.1007/s00520-025-09261-8)
Supplement: Supplementary file 1 — Supplementary file1 (PDF 57 KB) [file 520_2025_9261_MOESM1_ESM.pdf]

Barriers and Enablers to Prehabilitation prior to Breast Cancer Surgery in a Regional Health Service - Patients

Barwon Health Services are interested in your views on the development of our pre-operative exercise and education service for breast cancer patients.

As someone who has been in the care of Barwon Health cancer services for breast cancer surgery, your views will help us better understand your preferences for how you perceive and receive pre-operative care from our staff. In turn, your responses will help us improve our care for breast cancer patients.

Participant Information and Consent

This research aims to find out the reasons why people with breast cancer may or may not participate in an exercise and education program (Prehabilitation) before they receive their breast surgery. It will help Barwon Health to implement Prehabilitation within the breast cancer services in the most effective way for patients, and make recommendations about how that service can be improved.

Recent research studies have shown that Prehabilitation can improve quality of life, strength, fitness, mood, tolerance of cancer treatment, reduce hospital stay and aid quicker recovery . Exercise with a trained professional has been shown to be safe and effective for people living with cancer. Developing services appropriately therefore has the potential to improve the treatment outcomes and quality of life for people with breast cancer in our region.

The project will run for 12 months, to allow us to collect opinions from a wide range of service users. In addition to service improvements we aim to publish our findings in a peer-reviewed publication and via presentations within and external to Barwon Health

Eligibility

You are eligible to take part in this project if you meet the following criteria:

Age 18+ years and able to consent independentlyDiagnosis of breast cancer for which you have received surgeryAttending Barwon Health for your cancer treatment

What are we asking from you?

Information about this project is being distributed to patients who have undergone cancer-related breast surgery by the ward physiotherapist and via poster on the post-operative wards.

To participate in this project we are asking you to complete a one-off survey.. The online version of this survey is available as part of this link. Completing the survey will take 5 to 10 minutes.

If you would like a paper copy of the survey, please contact the research team or your ward physiotherapist who will contact us on your behalf.

Do I have to participate?

Your decision to take part in this survey is voluntary, your care will not be affected.

Please read this information carefully before making your decision. We are happy to answer any further questions you may have.

What are the possible risks of participating?

Apart from the time it will take you to complete the survey, we do not foresee any risks to you. The questions you will be asked are unlikely to cause any distress. If you do feel any concerns having completed the survey, please seek support from your cancer care team.

Your survey responses are anonymous and you will not be identified in any reporting of results. If you choose to complete a paper version of the survey, we will provide you with a SAE to return the anonymous survey to the research team. Your hard-copy responses will the copied onto the secure electronic data base by a member of the research team.

There will be no direct benefits to you, but it is intended that the data that we collect will assist with improving the quality of patient care services at Barwon Health.

Data Management

There will be no codes or information on the survey that will enable investigators to identify you. You are not required to provide your name on the survey. Returned surveys will be stored on the secure RedCap survey platform, and any computer files from this study will be password protected. This data will be destroyed after seven years. You are also invited to contact the Principal Investigator, using the above details, with further comments or to obtain a copy of the findings.

Can I change my mind after I have completed the survey?

Your survey responses are anonymous. This means that once the survey is submitted, we are unable to identify and withdraw your data.

Who has reviewed the research project?

The project has been approved by the Human Research Ethics Committee of Barwon Health, and will be carried out according to the National Statement on Ethical Conduct in Human Research (2007). This statement has been developed to protect the interests of people who agree to participate in human research studies.

Who can I contact?

If you have any questions, or would like further information about this project, please contact the Site Principal Investigator April Chiu.

E: april.chiu@barwonhealth.org.au

P: 03 4315 3830

If you have any concerns and/or complaints about the project, the way it is being conducted, or your rights as a participant, and would like to speak to someone independent of the project, please contact: Barwon Health Human Research Ethics Committee.

E: REGI@barwonhealth.org.au

P: 03 4215 3372

Please reference the Study ID Number: 22/38

If you do not wish to participate, you may tick ‘No, I do not consent to participate’. If you decide you want to take part in the survey please tick ‘Yes, I consent to participate’ and proceed through the survey.

If you tick ‘Yes’, you are telling us that you:

Understand what you have readConsent to take part in the pilot programConsent to have the tests and treatments that are describedConsent to the use of your personal and health information as described.

Thank you for taking the time to read this information.

To start the survey, please tick 'Yes, I consent to participate' and proceed through the survey.	◯ Yes - I consent to participate<br>◯ No - I do not consent to participate
Demographics - What is your current age?	◯ 18-24<br>◯ 25-34<br>◯ 35-44<br>◯ 45-54<br>◯ 55-64<br>◯ 65+yrs

Location: How far away do you live from the Barwon Health Sunrise Centre - main location of the Oncology Rehabilitation Program? (GMHBA stadium 370 Moorabool Street, South Geelong)	◯ 0-5km<br>◯ 5-10km<br>◯ 20-30km<br>◯ 30-40km<br>◯ 40-50km<br>◯ >50km
Demographics - What gender do you identify as?	◯ Male<br>◯ Female<br>◯ Non-binary<br>◯ Prefer not to answer
Demographics - Gender<br>I use a different term (please specify):	__________________________________
Demographics - Language<br>What is the MAIN language spoken at your home?	◯ English<br>◯ Other (please specify)<br>◯ I prefer not to answer
Demographics - Are you of Aboriginal and/or Torres Strait Islander background?	◯ Yes<br>◯ No<br>◯ I prefer not to answer
Demographics - Do you identify as part of the LGBTQIA+ community?	◯ Yes<br>◯ No<br>◯ I prefer not to answer
Did you participate in an oncology specific exercise and education program prior to your surgery?	◯ Yes<br>◯ No
Prior to your surgery, were you completing any regular structured exercise (at lease once a week)?	◯ Yes<br>◯ No<br>◯ I prefer not to answer
Prior to your surgery, what duration of exercise did you regularly complete?	◯ < 30 mins per week<br>◯ 30-60 mins per week<br>◯ > 60mins per week<br>◯ Further comments

Perceptions of exercise-based appointments

Do you remember being offered the opportunity to attend physiotherapy/exercise physiology BEFORE your breast surgery?

- ◯ Yes
- ◯ No
- ◯ I prefer not to answer

Did you attend physiotherapy/exercise physiology at Barwon Health BEFORE your breast surgery?

- ◯ Yes
- ◯ No
- ◯ I prefer not to answer

How did you attend your physiotherapy/exercise physiology appointments before surgery? Tick all that apply

- ☐ Face to face
- ☐ Phone
- ☐ Video Call (Healthdirect or Webex)

Did you find physiotherapy/exercise physiology before surgery helpful?

- ◯ Yes
- ◯ No
- ◯ I prefer not to say

If yes, why was it helpful?

____

If no, why was it not helpful?

____

How likely is it that you would recommend pre-operative exercise to another cancer patient?

0 - Very Unlikely — 5 - Very Likely

(Place a mark on the scale above)

Exercise forms a key part of a prehabilitation programme. We would like to improve our understanding of the things that might influence your motivation to undertake exercise prior to surgery.
Please indicate the extent to which you agree or disagree that each item kept you from exercising before surgery

	Strongly disagree	Disagree	Neither agree not disagree	Agree	Strongly Agree
1. Lack of convenient facilities	○	○	○	○	○
2. Fear of injury	○	○	○	○	○
3. Exercise not enjoyable	○	○	○	○	○
4. Cancer or treatment-related symptoms (pain, joint stiffness, nausea, other)	○	○	○	○	○
5. Doctor's recommendation not to exercise	○	○	○	○	○
6. Self-consciousness or embarrassment	○	○	○	○	○
8. Unpleasant sensation or symptoms caused by exercise	○	○	○	○	○
9. No instructor to guide me/do not know how to exercise	○	○	○	○	○
11. Fear of making the cancer or treatment-related symptoms worse	○	○	○	○	○
12. Lack of support from others	○	○	○	○	○
13. Too much information to process after breast cancer diagnosis	○	○	○	○	○
14. Exercise not important to me	○	○	○	○	○
15. Weather conditions	○	○	○	○	○
18. Lack of interest	○	○	○	○	○
19. Transportation problems	○	○	○	○	○
22. Lack of equipment or proper clothing	○	○	○	○	○
23. Fear of making other health problems worse	○	○	○	○	○
24. Financial costs/fees	○	○	○	○	○
25. Live too far away from exercise/health facilities	○	○	○	○	○
26. Other preferences for leisure activities	○	○	○	○	○
27. No one to exercise with	○	○	○	○	○

28. Fatigue - too tired	◯	◯	◯	◯	◯
29. Lack of time	◯	◯	◯	◯	◯
30. Having been diagnosed with cancer	◯	◯	◯	◯	◯
31. No safe place to exercise	◯	◯	◯	◯	◯
32. Lack of motivation	◯	◯	◯	◯	◯
33. Social or family responsibilities	◯	◯	◯	◯	◯
34. Did not see the need to exercise	◯	◯	◯	◯	◯

If you have any further information you are able to share with us about exercise after your diagnosis and prior to your surgery, please add your comments here.

____

If it was offered to you prior to surgery, would you have been interested in participating in a tailored exercise and education program for people with breast cancer in the time leading up to your surgery?

◯ Yes
◯ No

Please indicate why you would be interested. Tick all that apply

☐ My doctor told me to
☐ Personal fitness benefits
☐ Mental Health reasons
☐ Heard from others that it is beneficial
☐ To learn more about how I can be prepared for surgery

Do you think any of the following would be useful to patients when they have their first contact with the breast cancer clinic? Tick all that apply

☐ Written information about exercise and breast cancer
☐ Online info (website, video etc) about exercise and breast cancer
☐ Written info about pre-operative exercise and education services
☐ The ability to self-refer to prehabilitation (exercise + education) services
☐ Breast cancer exercise related app for my phone
☐ Access to remote Physiotherapy/Exercise appointment prior to surgery
☐ Access to face-to-face Physiotherapy/Exercise appointment prior to surgery

---

What was your preferred method of interaction with the Oncology Rehabilitation Service?

☐ Phone ☐ Telehealth (videocall)
☐ Face-to-face (Sunrise Centre)
☐ Email

Do you have any other feedback or suggestions for ways to improve our pre-operative exercise service for breast cancer patients?

____

Would you like to receive a copy of the study findings at the conclusion of the research project?

◯ Yes - proceed to collection of contact details for study findings
◯ No - end the survey
