# Supplementary material for: Barriers and enablers to exercise prehabilitation before breast cancer surgery in an Australian regional health service: patient and clinician perspective
Source: Support Care Cancer. 2025 Feb 21;33(3):211. doi: 10.1007/s00520-025-09261-8 (PMC11845435; doi:10.1007/s00520-025-09261-8)
Supplement: Supplementary file 2 — Supplementary file2 (PDF 52 KB) [file 520_2025_9261_MOESM2_ESM.pdf]

Barriers and Enablers to Prehabilitation prior to Breast Cancer Surgery in a Regional Health Service - Clinicians

Barwon Health Services are interested in your views on the development of our pre-operative exercise and education service for patients with breast cancer.

As someone who provides care as part of Barwon Health cancer services for breast cancer surgery, your views will help us better understand how you perceive pre-operative rehabilitation services. In turn, your responses will help us improve our care for breast cancer patients.

Participant Information and Consent

This research aims to explore the reasons why people with breast cancer may or may not participate in an exercise and education program (Prehabilitation) before they receive their breast surgery. It will help Barwon Health implement breast cancer services in the most effective way for clinicians and patients, and make recommendations about how that service can be improved. Effective service implementation has the potential to improve treatment outcomes and quality of life for people with cancer in our region.

Recent research studies have shown that Prehabilitation can improve quality of life, strength, fitness, mood, tolerance of cancer treatment, reduce hospital stay and aid quicker recovery. Exercise with a trained professional has been shown to be safe and effective for people living with cancer. Developing services appropriately therefore has the potential to improve the treatment outcomes and quality of life for people with breast cancer in our region.

The project will run for 12 months, to allow us to collect opinions from a wide range of service users. In addition to service improvements we aim to publish our findings in a peer-reviewed publication and via presentations within and external to Barwon Health

Eligibility

You will be eligible to take part in this project if you meet the following criteria:

Age 18+ years and able to consent independentlyHealth professional working at Barwon HealthCurrently working with breast cancer patients prior to surgical intervention

What are we asking from you?

As part of this project, we are asking clinicians working in breast cancer services to fill out a one-off survey. We would be grateful if you could take 5-10 minutes to complete the survey that follows. Completion and return of the survey will be understood to imply that you have given informed consent to participate in this research project.

Do I have to participate?

Your decision to take part in this survey is voluntary.

Please read this information carefully before making your decision. We are happy to answer any further questions you may have.

What are the possible risks of participating?

Apart from the time it will take you to complete the survey, we do not foresee any risks to you. The questions you will be asked are unlikely to cause any distress.

Your survey responses are anonymous and you will not be identified in any reporting of results. If you choose to complete a paper version of the survey, we will provide you with a SAE to return the anonymous survey to the research team. Your hard-copy responses will the copied onto the secure electronic data base by a member of the research team.

There will be no direct benefits to you, but it is intended that the data that we collect will assist with improving the quality of patient care services at Barwon Health.

Data Management

There will be no codes or information on the survey that will enable investigators to identify you. You are not required to provide your name on the survey. Returned surveys will be stored on the secure RedCap survey platform, and any computer files from this study will be password protected. This data will be destroyed after seven years. You are also invited to contact the Principal Investigator, using the above details, with further comments or to obtain a

copy of the findings.

Can I change my mind after I have completed the survey?

Your survey responses are anonymous. This means that once the survey is submitted, we are unable to identify and withdraw your data.

Who has reviewed the research project?

The project has been approved by the Human Research Ethics Committee of Barwon Health, and will be carried out according to the National Statement on Ethical Conduct in Human Research (2007). This statement has been developed to protect the interests of people who agree to participate in human research studies.

Who can I contact?

If you have any questions, or would like further information about this project, please contact the Site Principal Investigator April Chiu.

E: april.chiu@barwonhealth.org.au

P: 03 4315 3830

If you have any concerns and/or complaints about the project, the way it is being conducted, or your rights as a participant, and would like to speak to someone independent of the project, please contact: Barwon Health Human Research Ethics Committee.

E: REGI@barwonhealth.org.au

P: 03 4215 3372

Please reference the Study ID Number: 22/38

If you do not wish to participate, you may tick 'No, I do not consent to participate'. If you decide you want to take part in the survey please tick 'Yes, I consent to participate' and proceed through the survey.

If you tick 'Yes', you are telling us that you:

Understand what you have readConsent to take part in the pilot programConsent to have the tests and treatments that are describedConsent to the use of your personal and health information as described.

We appreciate your time and insights.

Thank you.

To start the survey, please tick 'Yes, I consent to participate' and proceed through the survey.	◯ Yes - I consent to participate<br>◯ No - I do not consent to participate
What is your PRIMARY clinical role in the care of breast care patients planned for surgery?	◯ Breast Care Nurse<br>◯ Surgeon<br>◯ Medical Oncologist<br>◯ Radiation Oncologist<br>◯ Allied Health<br>◯ Registrar (Surgical)<br>◯ Registrar (Medical Oncology)<br>◯ Intern (Surgical)<br>◯ Intern (Medical Oncology)<br>◯ Other
Please identify your clinical role:	________________________________

How long have you been working in the field of oncology?	◯ 0 - 2 years<br>◯ 2 - 5 years<br>◯ 5 - 10 years<br>◯ 10 - 15 years<br>◯ 15 - 20 years<br>◯ 20+ years
2. Please select the type of cancer(s) that is/are your main area(s) of clinical focus:	☐ Breast Cancer<br>☐ Prostate Cancer<br>☐ Cancers of the CNS<br>☐ Gastrointestinal cancers<br>☐ Genitourinary cancers<br>☐ Gynaecological cancers<br>☐ Head and neck cancer<br>☐ Haemotological cancers<br>☐ Lung cancer<br>☐ Melanoma and skin cancers<br>☐ Other

Barriers to Exercise Recommendations

The following is a list of currently published BARRIERS to discussion about exercise or referral to an exercise program. For each, please indicate how strongly you agree or disagree with each of the statements below, as relevant to your current practice

	Strongly Disagree	Disagree	Neither disagree or agree	Agree	Strongly Agree
My training does not qualify me to discuss exercise for breast cancer or refer to an exercise program	◯	◯	◯	◯	◯
I perceive pre-operative exercise and education to lack relevance to my patients' breast cancer or symptoms	◯	◯	◯	◯	◯
I feel that there are situations in patients with breast cancer that I treat where exercise would be unsafe	◯	◯	◯	◯	◯
There is limited time during a pre-operative patient visit to discuss exercise in breast cancer	◯	◯	◯	◯	◯
I do not have the knowledge on how or where to refer a patient to pre-operative breast cancer exercise and education	◯	◯	◯	◯	◯
It feels futile to recommend exercise to a patient with breast cancer I know has a poor prognosis	◯	◯	◯	◯	◯
I feel that referring a patient with breast cancer before their surgery for exercise will make him/her feel guilty for not having done exercise prior to their cancer diagnosis	◯	◯	◯	◯	◯
Patients have been told by other health care providers, friends, or family to rest	◯	◯	◯	◯	◯
I know that a patient has refused other support services in the past	◯	◯	◯	◯	◯

I am not convinced of the literature with respect to pre-operative exercise and breast cancer outcomes	○	○	○	○	○
I feel there is insufficient time for the patient to participate in meaningful exercise and education prior to their breast surgery	○	○	○	○	○

Please add further comments regarding other barriers that you feel are relevant: ______________________________

The following is a list of currently published FACILITATORS to discussion about exercise or referral to an exercise program. Please indicate how helpful each would be in providing exercise counselling/referral in your current practice:

	Strongly Disagree	Disagree	Neither disagree or agree	Agree	Strongly Agree
Written information about exercise in cancer (indications, guidelines, referral process, & safety information)	◯	◯	◯	◯	◯
Clinician education session about exercise in patients with cancer (indications, guidelines, referral process, & safety information)	◯	◯	◯	◯	◯
PAPER form/prescription pad with referral information	◯	◯	◯	◯	◯
ELECTRONIC/WEB-BASED form/prescription pad with referral information	◯	◯	◯	◯	◯
AUTOMATIC paper or electronic referral process	◯	◯	◯	◯	◯
Posters for patients to see so they can ask about exercise of their own accord	◯	◯	◯	◯	◯
For this information to be provided to patients outside of the clinic time I have to see the patient	◯	◯	◯	◯	◯
For this information to be available as a patient handout	◯	◯	◯	◯	◯
Having a physiotherapist or exercise physiologist available as part of the clinical team	◯	◯	◯	◯	◯
Having a physiotherapist or exercise physiologist included in the preoperative appointments prior to surgery	◯	◯	◯	◯	◯

Please add any further thoughts you have here about potential facilitators in this process: ______________________________

Would you like to receive a copy of the study findings at the conclusion of the research project?

◯ Yes - proceed to collection of contact details for study findings
◯ No - end the survey
